# How Sociodemographic Factors Impact the Utilization of Recommended Clinical Preventive Screening Services in Poland: A Nationwide Cross-Sectional Study

**DOI:** 10.3390/ijerph182413225

**Published:** 2021-12-15

**Authors:** Siddarth Agrawal, Sebastian Makuch, Gabriella Lachowicz, Mateusz Dróżdż, Krzysztof Dudek, Grzegorz Mazur

**Affiliations:** 1 Department and Clinic of Internal Medicine, Occupational Diseases, Hypertension and Clinical Oncology, Wroclaw Medical University, Borowska St. 213, 50-556 Wrocław, Poland; gabriella.lachowicz@umw.edu.pl (G.L.); grzegorz.mazur@umw.edu.pl (G.M.); 2 Department of Clinical and Experimental Pathology, Wroclaw Medical University, St. K. Marcinkowskiego 1, 50-368 Wrocław, Poland; sebastian.mk21@gmail.com; 3 Faculty of Medicine, Wroclaw Medical University, J. Mikulicza-Radeckiego 5, 50-345 Wrocław, Poland; mateuszdrozdz5208@gmail.com; 4 Faculty of Mechanical Engineering, Wroclaw University of Science and Technology, St. I. Łukasiewicza 5, 50-371 Wrocław, Poland; krzysztof.dudek@pwr.edu.pl

**Keywords:** sociodemographic factors, clinical preventive services, cardiovascular disease, cancer

## Abstract

Cardiovascular disease (CVD) and cancer are the most frequent causes of mortality in Poland. To date, no study in Poland has attempted to analyze the impact of sociodemographic factors on the utilization of all recommended preventive services for these diseases. To address this challenge, a nationwide cross-sectional study was conducted. One thousand adults aged 18 years or older were interviewed using computer-assisted telephone surveys conducted via random selection. A representative population was obtained in accordance with existing demographics per voivodeship in Poland. We assessed whether factors such as age, gender, body mass index (BMI), net income, household size, place of residence, and education impacted the odds ratio of utilizing recommended preventive services for CVD and cancer. We determined that elderly patients receive influenza vaccination, measure blood pressure, PSA concentration, glucose and lipid profiles, and undergo colonoscopy and mammography more often than younger counterparts. Men were more often influenza vaccinated (OR = 1.56, 95% CI: 1.07–2.27) than women, while women measured blood glucose more often than men (OR = 0.62, 95% CI: 0.42–0.93). Furthermore, net income < 2000 PLN, BMI < 24 kg/m^2^ and at least secondary education level were found to be crucial predictors of undergoing mammography (OR = 2.16; 95% CI: 1.26–3.72), cervical smear tests (OR = 1.99, 95% CI: 1.24–3.17), and lipid measurements (OR = 1.76, 95% CI: 1.07–2.91), respectively. Educating people and financial support seem to play a crucial role in implementing novel campaigns and preventive programs in Poland. Addressing each significant factor may be of paramount importance in improving the receipt of preventive services and warranting greater preventive care coverage in the Polish population.

## 1. Introduction

In 2018, cardiovascular disease (CVD) and cancer were the two most common causes of mortality in Poland, accounting for 40.5% and 24.5% of deaths, respectively [1]. Including CVD cases, this country continues to record a substantially higher mortality rate in comparison to other countries in the European Union (EU) [2]. This situation is also unfavorable for Polish inhabitants when it comes to cancer, but to a lesser extent than CVD. In Poland, trachea, bronchus, and lung cancer are the greatest risks of death, representing 23.4% of all deaths caused by malignant neoplasms. Other cancer types occurred much less frequently [2]. Nevertheless, due to such high mortality rates caused by CVD diseases and cancer, there is an urgent need to increase the utilization of preventive care services in the Polish population. Furthermore, due to the observed increase in population aging over time, these behaviors are particularly important nowadays. For instance, the current life expectancy in Poland in 2021 is 78.95 years, while 50 years ago, the life expectancy in this country was 69.79 years [3]. The utilization of preventive services as a strategy to decrease, delay, or prevent cancer and cardiovascular diseases may increase health expectancy and permit individuals to age gracefully for as long as possible [4].

Facing the challenge to intensify preventive measures, in 2016, the Council of Ministers implemented the Polish National Health Program 2016–2020. This essential document of the Polish health care system aims to improve diet, nutrition, and physical activity, prevent and reduce problems associated with psychoactive substances, addiction, etc., promote healthy and active aging, and contribute to improved reproductive health [5]. To ameliorate the health-related quality of life and reduce health inequalities, this program contains recommended clinical preventive services for CVD and malignant neoplasms. These easily accessible, free of charge, and covered by public funds tools are grouped into different subpopulations, according to the appropriate gender and age [6]. 

In our previous study, for the first time in Poland, we investigated the utilization of clinical preventive services in a publicly funded healthcare setting, including preventive screening and preventive counseling. Our findings represent an alarming situation; from a total of 1000 surveyed patients, only 6.4% (95% CI: 4.88, 7.92) had received all recommended preventive services [7]. By combining the results from our study and recommendations of the Polish National Health Program 2016–2020, we generated a list of preventive screening tests with a strong recommendation for individuals at risk of CVD and cancer (Table 1). Among tests for CVD, measurement of blood pressure, blood glucose, and lipid profile are considered strongly recommended. The most crucial examinations for cancer screening are cervical smear, mammography, colonoscopy, and PSA assessment. In addition, we also analyzed the utilization of flu vaccination and general practitioners’ (GP) visits, which play a significant role in initiating the proper treatment for both diseases. It is worth mentioning that flu vaccination and PSA assessment were the least frequently received screening tools, suggesting a lower strength of recommendation compared to the other preventive services [5].

In this study, we aimed to characterize the impact of sociodemographic factors on the utilization of clinical preventive screening tests recommended by the Polish National Health Program 2016–2020, with the highest strength of recommendations for CVD and cancer. Since different preventive services are recommended to different target groups depending on age and gender, we analyzed these services separately. Based on this approach, we identified groups of people that should be advised on CVD and cancer screening and actively encouraged to participate in preventive care. Furthermore, such data may be crucial in introducing targeted campaigns, incentives and appropriately modifying existing policies to reduce mortality and morbidity of CVD and cancer in Poland. 

## 2. Materials and Methods

### 2.1. Studied Population and Inclusion Criteria

A cross-sectional study was carried out in May–June 2020 in Poland. One thousand adults aged 18 years or older were interviewed using computer-assisted telephone surveys carried out using random selection. The response rate was 42%; from a total of 2381 calls, 1000 respondents were referred to as a representative sample of this study. We used proportionate stratified sampling depending on the demographic structure of voivodeships, the highest-level administrative division of Poland, to obtain a representative sample of the population. In addition, target quotas were set for age and gender strata in each geographical region. The proper size of the sample was calculated using the following formula:

(1) $$\mathrm{Sample}\;\mathrm{size}=\frac{Z_{1-a/2}{}^{2}p\left(1-p\right)}{d^{2}}$$

where $Z_{1-a/2}$ is the standard normal variate (at 5% type 1 error *p* < 0.05)—1.96; *p* is the expected prevalence obtained from *a* pilot study—0.7; and *d* is the absolute precision—0.0187.

### 2.2. Interviews

Verbal consent was obtained from participants prior to each interview. The average duration of the interview was 15 min. The following measures were enforced to ensure the high quality of interviews:

1. Interviewers were appropriately trained.
2. A data collection supervisor supervised each interview.
3. Interview recordings were evaluated at random by a study coordinator.
4. The transcripts were not returned to participants for comment and/or correction.
5. No repeat interviews were carried out.

### 2.3. Independent Variables

The following sociodemographic factors (independent variables) were considered: age, sex, body mass index (BMI) calculated from body height (cm) and mass (kg) of every participant (kg/m^2^), residence, household size, level of education, and net income per person per month (in Polish currency—PLN) in the household. Relevant potential predictors, such as place of residence, household size, education, and net income, were further divided into subcategories to facilitate data evaluation (village; town, less than 20,000 inhabitants; town, between 20,000 to 100,000 inhabitants; town, between 100,000 to 200,000 inhabitants; town, between 200,000 to 400,000 inhabitants; town, more than 400,000 inhabitants/lives alone; lives with a partner; lives with a partner and children; lives alone with children; lives with family; other situation/primary; vocational; secondary; higher education <500 PLN; 501–1000 PLN; 1001–2000 PLN; 2001–3000 PLN; more than 3000 PLN, respectively). Participants with incomplete data were excluded from the study; however, participants who refused to provide information about their income were included in the study.

### 2.4. Outcomes Assessed

The analysis focused on determining the influence of sociodemographic factors on the utilization of preventive screening tests for CVD and cancer with high strength of recommendation [7]. All recommended preventive screening tests for specific age and gender groups, as well as the reference period for each test, are listed in Table 1. 

Four main groups of outcomes were analyzed.

1. GP visit (in the previous 12 months—from May–June 2019 to May–June 2020).
2. Flu vaccination (in the previous 12 months—from May–June 2019 to May–June 2020).
3. CVD: glucose, lipid profile, and blood pressure (BP) measurement (appropriate to an age-sex group; in previous years; depending on a recommended reference period of each particular preventive service).
4. Cancer: colorectal, breast, cervical, and prostate cancer screening (appropriate to an age-sex group; in previous years; depending on a recommended reference period of each particular preventive service).

Participants were asked to state whether they had received appropriate services (outcomes) within the targeted period (as outlined in Table 1). Questions were designed to limit responses to “yes” or “no” answers. The study survey is available in Appendix A.

### 2.5. Statistical Analysis

We employed a composite measure to evaluate whether an individual received appropriate preventive service according to a specific age and gender group (as shown in Table 1). Statistical analysis was performed using Statistica v.13.3 (TIBCO Software Inc., Palo Alto, CA, USA). A logistic regression model was used. The selection of independent variables for the model was performed using backward stepwise regression. The model included variables that were significant in the univariate analysis at the level of *p* < 0.2. Ordinal variables (categorical, e.g., age group, size of the place of residence) were dichotomized. All variables taken into account in the logistic regression analysis were binary. The odds ratio and its 95% confidence intervals (95% CI) were estimated for each predictor. The statistical significance was determined using two-tailed *p* values and was reported at a *p* < 0.05 level.

### 2.6. Approval and Compensation

The study was approved by the bioethics committee at Wroclaw Medical University (Approval No. 142/2020.) Participants did not receive compensation for their involvement in the study.

## 3. Results

Out of the 1000 interviewed participants, 520 were female and 480 were male. The average age of respondents was 47 years (SD ± 17 years). Women were older than men by approximately 2.6 years (48.7 vs. 46.1 years; *p* = 0.011; Figure S1A). They raised children on their own more often than men (6.2% vs. 2.1%; *p* = 0.001, Figure S1B). In contrast, the male population lived more often with families (24.0 vs. 17.3; *p* = 0.009; Figure S1B). Only 54 people refused to share their net income. Men declared a slightly higher net income than women by approximately 157 PLN (2243 PLN vs. 2086 PLN; *p* = 0.005; Figure S1C). Furthermore, including the assumptions implemented by the World Health Organization (WHO) [8], 19 people were underweight (19/1000; 1.9%), 414 people were normal-weight (414/1000; 41.4%), 373 patients were overweight (31/1000; 37.3%), and 194 people were obese (194/1000; 19.4%). The body mass index (BMI) of men was higher than women by about 1.1 kg/m^2^ (26.8 kg/m^2^ vs. 25.7 kg/m^2^; *p* < 0.001; Figure S1D).

### 3.1. GP Visits and Influenza Vaccinations

One of the outcomes analyzed in this study was the frequency of GP visits. More common medical consultations certainly enhance awareness about potential risks for CVD and/or cancer, increasing the odds of participation in clinical preventive screening tests recommended by the Polish National Health Program 2016–2020. From a total of 1000 surveyed patients, 733 declared attending a GP visit for routine checkups, treatment control, and consultation caused by suspected health problems during the last 12 months. The remaining group reported less frequent GP visits. 154 patients had visited their GP in the previous 1 to 2 years, while 77 patients within 2 to 5 years and 25 patients more than 5 years ago. Moreover, 11 patients had never consulted with their GP. We indicated that the only independent predictor of visits to the GP in the last year was age. People aged 65 and over were medically consulted approximately 1.5 more often than those under 65 (OR = 1.55, 95% CI: 1.15–2.10). It is worth noting we did not determine the character of the visit to the GP, whether it was preventive (proactive, both as a part of secondary and tertiary prevention) or as a consequence of a health issue. However, it is recommended to visit a GP at least once every year, as GPs are responsible for providing preventive care to their patients during a visit, regardless of the character of the visit. Due to the nature of cross-sectional studies that have no dimension of time, they cannot support conclusions on causal relationships; hence, we could not address the causality issue in our study. 

Including all surveyed patients (*n* = 1000), only 128 (12.8%) were vaccinated against influenza in the last 12 months. Our findings show that (1) men (OR = 1.56, 95% CI: 1.07–2.27), (2) people over 65 (OR = 1.64, 95% CI: 1.08–2.50), and those who underwent medical consultation at least once within the last year (OR = 1.70, 95% CI: 1.06–2.72) received a flu vaccination more frequently. Furthermore, the chance of receiving the vaccination against influenza was more than 1.5 times higher among men (OR = 1.56, 95% CI: 1.07–2.27) than women. Detailed data on analyzing the impact of sociodemographic factors on the utilization of flu vaccination are presented in Table 2. 

### 3.2. Screening for CVD Risk Factors

Due to the high mortality rate caused by CVD in Poland, the Polish National Health Program 2016–2020 emphasizes preventive care. Including different clinical preventive services, glucose, lipid, and blood pressure (BP) measurement are characterized with the strongest recommendations for the Polish population [7]. The following analysis aims to determine the impact of sociodemographic factors on the utilization of preventive screening tests for CVD. We included appropriate target groups for each test in our statistical analysis. It is worth noting that appropriate age-sex groups with strong recommendations for preventive services for CVD were included in the analysis (Table 1). 

#### 3.2.1. Blood Pressure Testing

According to the Polish National Health Program 2016–2020 and data from our previous study [7], it is strongly recommended to measure the blood pressure of the whole Polish population at least annually, regardless of age or gender (Table 1). Thus, all interviewed patients (*n* = 1000) were asked if they had had their blood pressure checked by a healthcare professional in the last 12 months. We showed that 678 patients had had their blood pressure measured at least once within the last 12 months. The independent predictors of this preventive screening tool were (1) age over 65, (2) living with a partner and children, and (3) GP visits within the last 12 months. The odds ratio of performing blood pressure testing was approximately 1.5 times higher among the elderly population over 65 years old (OR = 1.61, 95% CI: 1.14–2.39) and those living with a partner and children (OR = 1.42; 95% CI: 1.06–1.90). Furthermore, blood pressure measurement increased nearly six times in people who visited a doctor at least once last year (OR = 6.23, 95% CI: 4.58–8.47). Detailed data on blood pressure measurement are presented in Table 3. 

#### 3.2.2. Blood Glucose Profile

According to the Polish National Health Program 2016–2020 and data from our previous study [7], it is strongly recommended that blood sugar measurement is carried out every three years among adults aged 45 to 69, regardless of gender (Table 1). From a total of 1000 interviewed participants, 488 aged 45–69 met the inclusion criteria, and 321 people answered affirmatively (321/488; 65.8% of the respondents, including 179 women and 142 men). We found that (1) women, (2) those aged over 50, (3) those living in towns with more than 100,000 inhabitants, (4) those living with a partner and children, and (5) those visiting a GP doctor at least once last year were more likely to undergo blood glucose measurement. The odds ratio of measuring the blood sugar level is more than three times higher in adults over 50 (OR = 3.23, 95% CI: 2.11–4.95) and more than 1.5 times higher among individuals living in cities with more than 100,000 inhabitants (OR = 1.61, 95% CI: 1.06–2.43) and people living with a partner and children (OR = 1.76, 95% CI: 1.16–2.68). Furthermore, the OR increased nearly twofold in people who had visited a GP at least once in the previous 12 months (OR = 2.37, 95% CI: 1.52–3.70) compared to those who had not. In addition, men underwent the test about 1.5 less often than women (OR = 0.62, 95% CI: 0.42–0.93). Detailed data are presented in Table 4.

#### 3.2.3. Lipid Profile

According to the Polish National Health Program 2016–2020 and data from our previous study [7], it is strongly recommended to undergo lipid measurements every five years for women aged 45 to 69 years and men aged 35 to 69 years (Table 1). From a total of 1000 interviewed people, 633 met the inclusion criteria (251 women aged 45–69 and 282 men aged 35–69). 365 people answered affirmatively (365/633; 57.7% of the respondents, including 163 women and 202 men). The primary individual predictors of lipid measurements were (1) age over 50, (2) living with families, (3) at least secondary level of education, and (4) at least one GP visit within the last year. The OR of lipid measurement was more than 4 times higher in people over 50 than younger participants (OR = 4.35, 95% CI: 3.05–6.20), approximately 2 times lower in patients living with families (OR = 0.56, 95% CI: 0.34–0.92), and 1.5 times higher in people with a secondary or higher level of education (OR = 1.76, 95% CI: 1.07–2.91). Furthermore, visiting a GP doctor increased the odds ratio of measuring lipid profiles nearly threefold (OR = 2.97, 95% CI: 2.00–4.42). Detailed data on analyzing interviewed patients who measured lipid profiles by health professionals are presented in Table 5. 

### 3.3. Screening for Cancer

Due to the alarming public health threat of cancer, it is critical to implement preventive screening tools to protect from this disease. According to the Polish National Health Program 2016–2020 and our previous data [7], we analyzed preventive screening tools with the strongest recommendations for appropriate age-sex groups. These tools are shown as follows: colonoscopy, mammography, cervical smear, and PSA measurement (Table 1). In the following analysis, we aim to determine the impact of sociodemographic factors on the utilization of preventive screening tests for different types of cancer. 

#### 3.3.1. Colonoscopy

To prevent the potential risk of colon cancer, adults are strongly recommended to have a colonoscopy, especially those aged 55 to 64 (according to the Polish National Health Program 2016–2020 and our previous study [7]). This screening tool should be applied at least every 10 years (Table 1). A total of 488 participants aged 55 to 64, including 251 women and 237 men, met the inclusion criteria. 93 adults answered affirmatively (93/488; 19.1% of respondents, including 57 women and 36 men), while the other group did utilize this screening tool (395/488; 80.9% of respondents, including 194 women and 201 men). Independent predictors of having a colonoscopy were age and the number of GP visits within the last 12 months. The odds ratio of undergoing a colonoscopy was approximately 2.5 times higher in adults over 60 than people below this age (OR = 2.56; 95% CI: 1.61–4.07) and 2 times higher in those who had visited a GP at least once during the previous year (OR = 2.18; 95% CI: 1.16–4.12). Detailed data on analyzing interviewed patients aged 55 to 64 who underwent a colonoscopy are presented in Table 6. 

#### 3.3.2. Mammography

A radiological method of breast examination—mammography—is strongly recommended for women to prevent breast carcinoma. This screening tool is suggested to be applied at least once in two years in women aged 50 to 69 years (Table 1). A total of 251 women met the inclusion criteria, and of these, 128 women answered affirmatively (128/251; 51.0%), while the remaining group did not utilize this screening tool (123/251; 49.0%). Independent predictors of mammography turned out to be age and net income. The odds ratio of undergoing mammography was approximately 5 times higher in women over 50 compared to women below this age (OR = 5.17, 95% CI: 2.87–9.33). Furthermore, women with a net income above 2000 PLN underwent mammography more often than those earning less than 2000 PLN (OR = 2.16; 95% CI: 1.26–3.72). Detailed data are presented in Table 7. 

#### 3.3.3. Cervical Smear

According to the Polish National Health Program 2016–2020 and data from our previous study [7], it is strongly recommended to undergo a cervical smear test at least once in 3 years in women aged 25 to 39 (Table 1). Out of 388 women who met the inclusion criteria, 260 answered affirmatively (268/388; 33%). The remaining did not undergo the test (128/388; 49%). Independent predictors of undergoing cervical smear test were (1) living with family, (2) lower body mass index, and (3) a visit to the doctor in the last year. The odds ratio of women living with their family undergoing a cervical smear test was 0.26 times lower than women living only with a partner, only with children, or with both partner and children (95% CI: 0.13–0.53). Furthermore, women with a BMI <24 kg/m^2^ and those who visited a GP doctor at least once in the previous year underwent cervical smear testing more often (OR = 1.99, 95% CI: 1.24–3.17 and OR = 2.18, 95% CI: 1.33–3.59). Detailed data are presented in Table 8. 

#### 3.3.4. PSA Assessment

Although PSA (prostate-specific antigen) assessment is the least utilized service among all cancer screening tools (126/480; 26.2%) [7], this method is recommended for men aged 50 to 69 (Table 1). Of 237 men, 62 underwent PSA measurement (62/237; 26.2%). Independent predictors of PSA assessment were (1) age over 60 years, (2) living in a city with a population of over 20,000 inhabitants, and (3) living with family. The odds ratio of measuring PSA concentration was approximately 5 times higher in men over 60 compared to those below this age (OR = 4.77; 95% CI: 2.49–9.14) and almost 2 times higher in men living in a city with a population of over 20,000 inhabitants (OR = 2.36; 95% CI: 1.10–5.04). Furthermore, men living with family are 4 times less likely to have their PSA measured than men living with others or alone (OR = 0.23; 95% CI: 0.06–0.80). Detailed data analyzing interviewed patients who had their PSA concentration measured by health professionals are presented in Table 9. 

## 4. Discussion

A variety of different reports evidenced the crucial correlation between health and sociodemographic status, including education, net income, employment and working conditions, lifestyle, and social support networks [9]. People with low social positions present at least twice the risk of developing severe disease and premature death [10]. This is observed when analyzing CVD and cancer mortality rates—two of the leading causes of death in Europe in recent years. For instance, in 2017, likely due to a distinct lifestyle and access to medicines, there was a 13-fold difference in female death rates from ischemic heart disease in France and Lithuania (32 deaths versus 429 per 100,000 women) and a 6-fold difference in male and female death rates from stroke in France and Bulgaria. Based on this observation, the authors of the European Cardiovascular Disease Statistics 2017 concluded that death rates from both ischemic heart disease and stroke were generally higher in low-developed countries and countries in political transition or reconstruction. Wide variations across the EU were also observed in cancer mortality. In 2016, the highest standardized death rates for cancer were recorded in Hungary and Croatia, each with rates of more than 330 per 100,000 inhabitants, while the lowest rate was recorded in Cyprus (194 per 100,000). These differences were explained by the gaps in the availability of cancer screening technology [11]. 

Social inequalities are observed in many dimensions: access to education, health care, clean water, food, security, natural resources, environmental protection, etc. [12]. Quality health care is possible only if individuals have appropriate access to education and a certain level of housing, occupation, and income, providing for their basic needs. All these factors, taken together, give a significant amount of autonomy to make decisions related to proper health care [13]. Taking into account Polish legislation, the National Health Program 2016–2020 aimed to “extend life, improve the life quality of the population, and reduce health inequalities.” Although this program turned out to be a non-effective tool in the significant improvement of the healthcare system, its implementation had a slightly positive effect on the achievement of suspected goals, increasing the life quality of the population, and reducing social inequalities in health. The reason for this observation was the lack of a clear definition of public health and imprecise determination of tasks implemented by the healthcare system. In this regard, the Supreme Chamber of Control in Poland requested that the definition of public health be precisely clarified to avoid further potential inaccuracies [11]. 

One of the significant functions of public health that is assumed to reduce social inequality is health promotion. It aims to improve the population’s health determinants, support health quality, social justice, and solidarity, and respect human rights. The task of health care providers is to systematically and constantly plan and implement activities and initiatives to promote health in local environments and build health awareness among the community [11]. The World Health Organization (WHO) considers this activity as one of the most effective in reducing health inequalities. Three areas of governmental actions need to be implemented:

1. Implementation and maintenance of a legal framework regulating and enabling actions to keep equalities in health.
2. Monitoring of health in different social groups, health effects of social inequalities, and results of activities aiming at reducing social inequalities, as well as their proper usage in the framework of conducted future interventions.
3. Providing the population with a fairer distribution of preventive screening tools with the promotion of human rights to health care, education, and decent housing.

A variety of different legislations and national programs promoting health allows participation in preventive screening without any costs. However, the observed low frequency of population willing to access preventive services is still a serious public health problem. Many people decide not to undergo preventive tests due to lack of time, awareness, or the presence of formal or psychological barriers (e.g., lack of insurance, fear of a diagnosis) [14]. Our cross-sectional study revealed that several clinical preventive tests were more frequently delivered (utilization greater than 50%), including the measurement of blood pressure (678/1000; 67.8%—Table 3), blood glucose (321/488; 65.8%—Table 4), and lipid profile (365/633, 57.5%—Table 5), as well as mammography (128/251; 51%—Table 7). However, other preventive tests, such as flu vaccination (128/1000; 12.8%—Table 2), colonoscopy (93/488; 19.1%—Table 6), and cervical smear (268/388; 33%—Table 8) require urgent attention. Higher delivery of these preventive services may be achieved through educating patients on the advantages of preemptive care. This may be implemented by telephone or online reminders that medical visits are vital to health maintenance, regular checkups can identify risk factors and problems before they become serious, and treatments are often more effective when the disease is caught relatively early [14]. These behaviors may reduce the potential risks of CVD or cancer.

Our results show the significance of medical consultations in the delivery of preventive care. The more frequently an individual visits a GP, the more likely a patient will utilize preventive services for CVD and cancer. GPs may positively influence the patient’s lifestyle choices and encourage them to take greater responsibility for their health, for example, by participating in preventive services [15]. According to our study, people who visited a GP at least once a year were more likely to receive a flu vaccination (OR = 1.70, 95% CI: 1.06–2.72—Table 2) and undergo blood pressure, (OR = 6.23, 95% CI: 4.58–8.47—Table 3), blood glucose (OR = 2.37, 95% CI: 1.52–3.70—Table 4), and lipid measurement (OR = 2.97, 95% CI: 2.00–4.42—Table 5), as well as undergo colonoscopy (OR = 2.18; 95% CI: 1.16–4.12—Table 6) and cervical smear testing (OR = 2.18, 95% CI: 1.33–3.59—Table 8). However, no statistical significance was observed in the case of mammography and PSA assessment. 

Age, as an unmodifiable factor, also plays a significant role in the formation and maintenance of inequalities in health. According to the National Institute of Public Health—National Institute of Hygiene in 2016, it was determined that Poles aged over 65 live in health for a shorter amount of time compared to average citizens of other EU members. The lives of younger people, men aged 10 to 44 and women aged 5 to 29, are primarily threatened by external causes, such as accidents, suicides, and the consequences of crime. In the following years, men are mainly at risk of CVD and, to a slightly lesser extent, cancer. In contrast, women’s lives up to 70 years are threatened primarily by cancer, which gives way to CVD in older age [16]. Therefore, due to the higher risk of CVD and cancer in elderly patients, there is a significant difference in utilizing preventive services between the young and older populations. For instance, Rotarou et al. performed a cross-sectional study to investigate preventive health services utilization rates for Chileans aged 15 years and over. Their statistical analysis revealed that older people had slightly higher use of preventive services than younger people [17]. This finding is consistent with our study, clearly illustrating that elderly patients had higher odds of adhering to the preventive recommendations. For instance, patients over 65 years were more likely to get an influenza vaccination (OR = 1.64, 95% CI: 1.08–2.5—Table 2). Furthermore, we determined that patients over 65 years were more likely to measure their blood pressure (OR = 1.65; 95% CI: 1.14–2.39—Table 3). Including the increasing risk of colon cancer in the older generation, patients over 60 years were more likely to have a colonoscopy (including adults aged 55 to 64 years; OR = 2.56, 95% CI: 1.61–4.07—Table 6). Moreover, women over 50 years were more likely to undergo mammography (OR = 5.17, 95% CI: 2.87–9.33—Table 7). In addition, men over 60 years had their serum prostate-specific antigen (PSA) concentration measured more often than younger respondents (including men aged 50 to 69 years; OR = 4.77, 95% CI: 2.49–9.14—Table 9). This examination plays a crucial role in the detection, diagnosis, and treatment monitoring of prostate cancer. It was determined that 86% of Polish male deaths caused by prostate cancer in 2014 occurred among men aged 65 and over. This percentage was slightly higher than the EU average [18]. Nevertheless, it is worth noting, these findings cover specific time frames (in years) depending on the particular analyzed preventive service, focused mainly on periods with strong recommendations, as suggested by findings from our previous study and data from Polish National Health Program 2016–2020. In this regard, our data did not reveal the situation of withdrawal from participating in preventive services at specific time frames, while these tests were potentially performed at a later age when the likelihood of suffering from CVD and/or cancer increases with age [19,20].

The incidences of influenza in Poland have increased drastically in the last 20 years, from less than 2 million cases in 2000 to more than 5 million cases in 2018 [21]. Fortunately, the mortality rate appears to be stable, with fewer than 200 deaths per annum. However, considering the aging population and increasing morbidity rates of influenza, it is undoubtedly beneficial to address these issues. One of the strategies aiming to reduce incidences of influenza is vaccination. Since 2010, the CDC’s Advisory Committee on Immunization Practices (ACIP) has recommended annual influenza vaccination of all healthy people aged ≥6 months, especially those who are at increased risk of suffering from the infection [22]. Nevertheless, since 2005, when only 8.6% of the Polish population received the flu vaccination, there has been a decreasing tendency to utilize this preventive service. In the fall of 2015–2016, only 6% received a flu vaccination, mainly residents of large cities, respondents earning at least PLN 2000, and patients over 65 years. Regardless of vaccination in this season, it was determined that young respondents aged 18–24 (51%), those earning at least PLN 2000 (45%), and residents of cities with more than half a million inhabitants (51%) had received the flu vaccination at least once in their lives. Furthermore, men were more willing to participate in this preventive service than women (40% vs. 28%) [23]. This result is consistent with our study showing that men were more willing to accept influenza vaccines than their female counterparts (OR = 1.56, 95% CI: 1.07–2.27—Table 2). One of the potential causes of this discrepancy was the conviction that females develop higher antibody responses and show greater vaccine efficacy than males. Furthermore, women experience more adverse side effects post-vaccination, including fever, pain, and inflammation [24]. Including the diverse array of Food Drug Administration (FDA, Silver Spring, MD, USA)-approved influenza vaccines available and the evidence of sex-specific responses to influenza infection, we propose that national vaccination campaigns should design vaccines to the individual’s biological sex. This conviction is a potential way to increase vaccination. Furthermore, this strategy may be applied to other vaccines for which sex differences in antibody responses and adverse reactions are reported (e.g., hepatitis A, B, diphtheria, pertussis, or anthrax) [25,26].

It is widely accepted that higher incomes—and other markers of socioeconomic circumstances—are associated with better health at the individual level. This relationship is found in morbidity outcomes for CVD and cancer. Income inequality rose markedly in wealthy nations starting in the 1970s. For instance, in the US, income inequality has increased by over 20 to 30% in 50 years [27]. The consequences of income inequalities are thought to be observed in the differences in the benefits and costs of higher education, the distribution of public goods, and the uneven diffusion of health innovations between rich and poor populations [28]. For instance, by earning more, we spend more on education and a healthy diet. Our study revealed that women aged 50 to 69 were more likely to undergo mammography if their monthly net income was at least PLN 2000 (OR = 2.16; 95% CI: 1.26–3.72—Table 7). This result is consistent with a cohort study by Williams et al. conducted in the United States [29]. One of the potential causes of these economic disparities may be the differential access to supplemental insurance, the method of communication and approach to individuals by health care providers, access to health care, and transportation costs [28]. All these factors should be considered during the implementation of subsequent national preventive programs, especially for women with reduced values of assets. Financial support for these women is essential, especially in current times, when increasing mortality and morbidity of breast cancer in Poland is observed [30]. 

Furthermore, our study revealed that women with a BMI < 24 kg/m^2^ underwent cervical smear testing more often (OR = 1.99, 95% CI: 1.24–3.17). Although obese women experience higher mortality from this disease, they undergo cervical smear testing less frequently than their counterparts with a BMI within the normal range. This statement seems to result from potential barriers to Pap testing for overweight and obese women. For instance, they often delay medical care due to a negative body image, embarrassment, a perceived lack of respect from health care providers, and avoidance of unwanted weight loss advice [31]. 

Prolonging the life expectancy of any population is an important marker of societal health. However, campaigns must emphasize the importance of improving healthy life years (HLY) and diminishing limitations in activities of daily living (ADL). The difference between Poland and the rest of the member states of the EU is stark. Only 31% of Poles above 65 have no chronic diseases (compared to 46% of the generalized EU population [2]). Furthermore, 23% of Poles over the age of 65 are reported to have limitations in ADL, compared to 18% of Europeans elsewhere [2]. Improvement in the population’s health and quality of life will lighten a load of an overburdened healthcare system in the long term and improve productivity by inherently giving more members of society the possibility of contributing to the economy.

### Limitations

The following limitations should be mentioned:

1. Data was not obtained from medical documentation. Respondents recalled answers to questions. These answers may be subject to recall bias. This increases the risk of overreporting the rate of utilization of various services.
2. Sociodemographic factors included in the survey were limited. There are, however, numerous other independent variables that could potentially influence the results.
3. To accurately represent the Polish adult population in our data, a stratified sampling per the voivodeships’ demographic structure was used. However, target quotas for sex and age strata were implemented in each geographical region. Therefore, we are aware of the presence of the inherent limitations of quota sampling.
4. The data collection took place during the outbreak of the COVID-19 pandemic (May–June 2020). Due to the threat of a rapid spread of the SARS-CoV-2 virus, which began to take its toll from the beginning of January 2020, many preventive programs in Poland have been partially or completely stopped. Considering our study, it is impossible to determine how the COVID-19 pandemic affects the final results. We cannot determine how much our results would have been different if there had been no pandemic. Nevertheless, we declare that considering GP visits, we have also included online medical consultations that were implemented by the Polish government to limit the spread of the virus. Furthermore, for the same reason, many Polish inhabitants lost their jobs or worked remotely, reducing the amount of a month’s salary and, consequently, affecting the final results of this study.

## 5. Conclusions

The receipt of preventive services in Poland is exceptionally low compared to the other EU countries. Our study identified sociodemographic factors, especially age, gender, net income, education, and BMI, as potentially influencing participation in preventive testing for cardiovascular diseases and cancer. Elderly patients were more likely to receive influenza vaccination and undergo blood pressure, glucose, lipid profiles, and PSA testing, as well as mammography and colonoscopy. Furthermore, men more often received influenza vaccination than women (OR = 1.56, 95% CI: 1.07–2.27), while women measured blood glucose more often than men (OR = 0.62, 95% CI: 0.42–0.93). Moreover, women with a net income above 2000 PLN and BMI <24 kg/m^2^ underwent mammography (OR = 2.16; 95% CI: 1.26–3.72) and cervical smear (OR = 1.99, 95% CI: 1.24–3.17) more often, respectively. In addition, lipid measurement was performed approximately 1.5 times more often in people with a secondary or higher level of education (OR = 1.76, 95% CI: 1.07–2.91). Addressing each of the significant factors may be of paramount importance in improving the utilization of these services, as recommended by the European Council. We believe our findings will be helpful for the implementation of campaigns and preventive programs for those who do not access preventive services despite strong recommendations by their GPs. Furthermore, considering the aging population and the rising morbidities associated with age, it may be vital to emphasize the importance of healthy life years in motivating society members to undergo preventative screening.

## Figures and Tables

**Table 1:** Prophylactic services (outcomes) assessed in the study, with screening recommendations relevant for the Polish population (according to the national recommendations for preventive screening, as summarized in Agrawal et al. [7]). All preventive services are publicly financed and available to all insured patients in appropriate age-sex groups.

	Preventive Services	Reference Period	Target Group
**General care**	General practitioner (GP) visit	Annual	All
	Influenza vaccination	Annual	All
**Cardiovascular risk factors**	Blood pressure	Annual	All
	Blood sugar	Every three years	Adults aged 45 to 69
	Lipid profile	Every five years	Females aged 45 to 69 men aged 35 to 69
**Cancer risk factors**	Colonoscopy	Every ten years	Adults aged 55 to 64
	Mammography	Every two years	Females aged 50 to 69
	Cervical smear	Every three years	Females aged 25 to 39
	PSA measurement	Annual	Males aged 50 to 69

**Table 2:** Results of univariate and multivariate logistic regression regarding sociodemographic factors associated with being vaccinated against influenza in the last 12 months, as well as the odds ratio (OR) and its 95% confidence interval (CI). (in red: the most statistically significant (*p*-value < 0.05) predictors of getting influenza vaccination in surveyed patients).

Predictors	Univariate Analysis	Multivariate Analysis	OR (95% CI)
	*p*	*p*	
Gender (male)	0.046	0.022	1.56 (1.07–2.27)
Age ≥ 65 years (yes)	0.030	0.021	1.64 (1.08–2.50)
Place of residence (>400,000 inhabitants)	0.359	-	-
Lives alone (yes)	0.826	-	-
Lives with a partner (yes)	0.483	-	-
Lives with a partner and children (yes)	0.709	-	-
Lives alone with children (yes)	0.518	-	-
Lives with the family (yes)	0.680	-	-
Level of education (higher)	0.190	>0.05	-
Net income ≥ 3000 PLN	0.187	>0.05	-
BMI < 25 kg/m^2^	0.839	-	
A visit to the family doctor in the last year (yes)	0.031	0.027	1.70 (1.06–2.72)

**Table 3:** Results of univariate and multivariate logistic regression regarding sociodemographic factors associated with blood pressure measurement as well as the odds ratio (OR) and its 95% confidence interval (CI) (in red: the most statistically significant (*p*-value < 0.05) predictors of performing blood pressure testing in surveyed patients).

Predictors	Univariate Analysis	Multivariate Analysis	OR (95% CI)
	*p*	*p*	
Gender (male)	0.940	-	-
Age ≥ 65 years (yes)	0.008	0.009	1.65 (1.14–2.39)
Place of residence (>20,000 inhabitants)	0.126	>0.05	-
Lives alone (yes)	0.028	>0.05	-
Lives with a partner (yes)	0.783	-	-
Lives with a partner and children (yes)	0.015	0.018	1.42 (1.06–1.90)
Lives alone with children (yes)	0.872	-	-
Lives with the family (yes)	0.242	-	-
Level of education (higher)	0.951	-	-
Income ≥ 1000 PLN	0.051	>0.05	-
BMI < 25 kg/m^2^	0.161	>0.05	-
A visit to the family doctor in the last year (yes)	<0.001	<0.001	6.23 (4.58–8.47)

**Table 4:** Results of univariate and multivariate logistic regression regarding sociodemographic factors associated with blood glucose measurement as well as the odds ratio (OR) and its 95% confidence interval (CI) (in red: the most statistically significant (*p*-value < 0.05) predictors of performing blood glucose profile within the last 3 years in surveyed patients).

Predictors	Univariate Analysis	Multivariate Analysis	OR (95% CI)
	*p*	*p*	
Gender (male)	0.008	0.021	0.62 (0.42–0.93)
Age ≥ 50 years (yes)	<0.001	<0.001	3.23 (2.11–4.95)
Place of residence (>100,000 inhabitants)	0.023	0.025	1.61 (1.06–2.43)
Lives alone (yes)	0.630	-	-
Lives with a partner (yes)	0.883	-	-
Lives with a partner and children (yes)	0.174	0.008	1.76 (1.16–2.68)
Lives alone with children (yes)	0.788	-	-
Lives with the family (yes)	0.095	>0.05	-
Level of education (secondary or higher)	0.067	>0.05	-
Income ≥ 5000 PLN	0.328	-	-
BMI < 25 kg/m^2^	0.587	-	-
A visit to the family doctor in the last year (yes)	<0.001	<0.001	2.37 (1.52–3.70)

**Table 5:** Results of univariate and multivariate logistic regression regarding sociodemographic factors associated with lipid measurement as well as the odds ratio (OR) and its 95% confidence interval (CI) (in red: the most statistically significant (*p*-value < 0.05) predictors of performing the lipid measurements within the last 5 years in surveyed patients).

Predictors	Univariate Analysis	Multivariate Analysis	OR (95% CI)
	*p*	*p*	
Gender (male)	0.003	>0.05	-
Age ≥ 50 years (yes)	<0.001	<0.001	4.35 (3.05–6.20)
Place of residence (>400,000 inhabitants)	0.039	>0.05	-
Lives alone (yes)	0.946	-	-
Lives with a partner (yes)	0.271	-	-
Lives with a partner and children (yes)	0.464	-	-
Lives alone with children (yes)	0.122	>0.05	-
Lives with the family (yes)	0.001	0.023	0.56 (0.34–0.92)
Level of education (secondary or higher)	0.032	0.027	1.76 (1.07–2.91)
Income ≥ 1000 PLN	0.375	-	-
BMI < 25 kg/m^2^	0.007	>0.05	-
A visit to the family doctor in the last year (yes)	<0.001	<0.001	2.97 (2.00–4.42)

**Table 6:** Results of univariate and multivariate logistic regression regarding sociodemographic factors associated with colonoscopy utilization as well as the odds ratio (OR) and its 95% confidence interval (CI) (in red: the most statistically significant (*p*-value < 0.05) predictors of performing colonoscopy within the last 10 years in surveyed adults aged 55 to 64).

Predictors	Univariate Analysis	Multivariate Analysis	OR (95% CI)
	*p*	*p*	
Gender (male)	0.036	>0.05	-
Age ≥ 60 years (yes)	<0.001	<0.001	2.56 (1.61–4.07)
Place of residence (>100,000 inhabitants)	0.109	>0.05	-
Lives alone (yes)	0.110	>0.05	-
Lives with a partner (yes)	0.166	>0.05	-
Lives with a partner and children (yes)	0.528	-	-
Lives alone with children (yes)	0.555	-	-
Lives with the family (yes)	0.059	>0.05	-
Level of education (higher)	0.642	-	-
Income ≥ 1000 PLN	0.375	-	-
BMI < 25 kg/m^2^	0.531	-	-
A visit to the family doctor in the last year (yes)	0.007	0.016	2.18 (1.16–4.12)

**Table 7:** Results of univariate and multivariate logistic regression regarding sociodemographic factors associated with mammography utilization as well as the odds ratio (OR) and its 95% confidence interval (CI) (in red: the most statistically significant (*p*-value < 0.05) predictors of performing mammography within the last 2 years in surveyed women aged 50 to 69).

Predictors	Univariate Analysis	Multivariate Analysis	OR (95% CI)
	*p*	*p*	
Age ≥ 50 years (yes)	<0.001	<0.001	5.17 (2.87–9.33)
Place of residence (>100,000 inhabitants)	0.605	-	-
Lives alone (yes)	0.618	-	-
Lives with a partner (yes)	0.386	-	-
Lives with a partner and children (yes)	0.579	-	-
Lives alone with children (yes)	0.916	-	-
Lives with the family (yes)	0.852	-	-
Level of education (secondary or higher)	0.904	-	-
Income ≥ 2000 PLN	0.002	0.005	2.16 (1.26–3.72)
BMI < 25 kg/m^2^	0.942	-	-
A visit to the family doctor in the last year (yes)	0.064	>0.05	-

**Table 8:** Results of univariate and multivariate logistic regression regarding sociodemographic factors associated with the cervical smear testing as well as the odds ratio (OR) and its 95% confidence interval (CI) (in red: the most statistically significant (*p*-value < 0.05) predictors of performing cervical smear within the last 3 years in surveyed women aged 25 to 39).

Predictors	Univariate Analysis	Multivariate Analysis	OR (95% CI)
	*p*	*p*	
Age ≥ 35 years (yes)	0.086	>0.05	-
Place of residence (>20,000 inhabitants)	0.104	>0.05	-
Lives alone (yes)	0.584	-	-
Lives with a partner (yes)	0.871	-	-
Lives with a partner and children (yes)	0.008	>0.05	-
Lives alone with children (yes)	0.921	-	-
Lives with the family (yes)	<0.001	<0.001	0.26 (0.13–0.53)
Level of education (secondary or higher)	0.196	>0.05	-
Income ≥ 1000 PLN	0.521	-	-
BMI < 25 kg/m^2^	0.027	0.004	1.99 (1.24–3.17)
A visit to the family doctor in the last year (yes)	0.003	0.002	2.18 (1.33–3.59)

**Table 9:** Results of univariate and multivariate logistic regression regarding sociodemographic factors associated with measurement of PSA concentration as well as the odds ratio (OR) and its 95% confidence interval (CI) (in red: the most statistically significant (*p*-value < 0.05) predictors of measuring the concentration of PSA within the last 12 months in surveyed men).

Predictors	Univariate Analysis	Multivariate Analysis	OR (95% CI)
	*p*	*p*	
Age ≥ 60 years (yes)	<0.001	<0.001	4.77 (2.49–9.14)
Place of residence (>20,000 inhabitants)	0.017	0.027	2.36 (1.10–5.04)
Lives alone (yes)	0.292	-	-
Lives with a partner (yes)	0.096	>0.05	-
Lives with a partner and children (yes)	0.174	>0.05	-
Lives alone with children (yes)	0.939	-	-
Lives with the family (yes)	0.009	0.021	0.23 (0.06–0.80)
Level of education (secondary or higher)	0.566	-	-
Income ≥ 1000 PLN	0.082	>0.05	-
BMI < 25 kg/m^2^	0.671	-	-
A visit to the family doctor in the last year (yes)	0.044	>0.05	-

## Data Availability

The data presented in this study are available on request from the corresponding author. The data are not publicly available due to ethical considerations.

## Supplementary Materials

The following are available online at https://www.mdpi.com/article/10.3390/ijerph182413225/s1; Figure S1: (A) Age of the studied women and men and the significance test result (B). The number (percentage) of respondents in groups differing in gender and household size and the test results for structure indicators (C). Average net income per capita in a household, declared by women and men, and the result of the significance test. (D) Male and female body mass index and significance test result.

## Appendix A. Study Survey (Translated from Polish)

1. What is your weight (in kilograms)?

2. What is your height (in centimeters)?

3. When was the last time you visited a doctor for a health assessment, follow-up care for an ongoing problem, or a concern that you have about your health? Do not include emergency visits or hospitalizations.

- within the past 12 months
- within the past 1 to 2 years
- within the past 2 to 5 years
- more than five years ago
- never

4. Have you been vaccinated against the flu within the previous 12 months?

- Yes
- No

**Sex-specific**

**Women:**

5. Within the past 12 months, have you had your blood pressure checked by a doctor, nurse, or other health care professional?

- Yes
- No

6. Within the last three years, have you had a blood glucose test?

- Yes
- No

7. Within the past five years, have you had your blood lipid profile tested?

- Yes
- No

8. Within the past ten years, have you had a colonoscopy?

- Yes
- No

9. Within the last three years, have you had a cervical smear test?

- Yes
- No

10. Within the last two years, have you had mammography? A mammography is an x-ray taken only of the breast by a machine that presses against the breast.

- Yes
- No

**Men:**

11. Within the past 12 months, have you had your blood pressure checked by a doctor, nurse, or other health care professional?

- Yes
- No

12. Within the last three years, have you had a blood glucose test?

- Yes
- No

13. Within the past five years, have you had your blood lipid profile tested?

- Yes
- No

14. Within the past ten years, have you had a colonoscopy?

- Yes
- No

15. During the past 12 months, have you had a PSA test?

- Yes
- No
