# Supplementary material for: How Sociodemographic Factors Impact the Utilization of Recommended Clinical Preventive Screening Services in Poland: A Nationwide Cross-Sectional Study
Source: Int J Environ Res Public Health. 2021 Dec 15;18(24):13225. doi: 10.3390/ijerph182413225 (PMC8701877; doi:10.3390/ijerph182413225)
Supplement: Supplementary file 1 [file ijerph-18-13225-s001.zip › ijerph-1399970-supplementary.pdf]

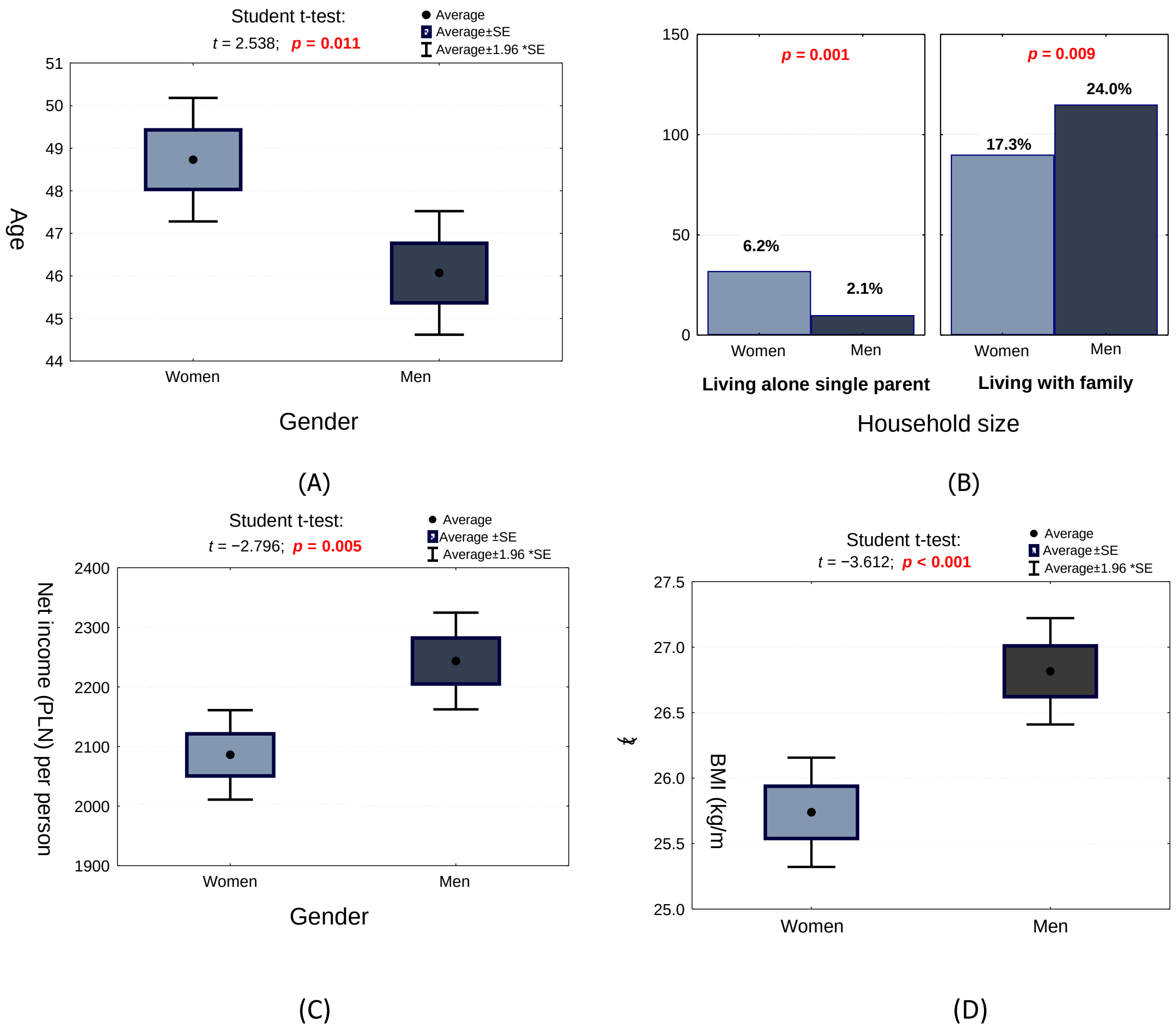

Figure S1. (**A**) Age of the studied women and men and the significance test result (**B**) The number (percentage) of respondents in groups differing in gender and household size and the test results for structure indicators, (**C**) Average net income per capita in a household, declared by women and men, and the result of the significance test result, (**D**) Male and female body mass index and significance test result
